# Implementation research: reactive mass vaccination with single-dose oral cholera vaccine, Zambia

**DOI:** 10.2471/BLT.16.189241

**Published:** 2017-10-19

**Authors:** Marc Poncin, Gideon Zulu, Caroline Voute, Eva Ferreras, Clara Mbwili Muleya, Kennedy Malama, Lorenzo Pezzoli, Jacob Mufunda, Hugues Robert, Florent Uzzeni, Francisco J Luquero, Elizabeth Chizema, Iza Ciglenecki

**Affiliations:** a Médecins sans Frontières, 78, rue de Lausanne, Case Postale 1016, 1211 Geneva, Switzerland.; b Republic of Zambia Ministry of Health, Lusaka, Zambia.; c Epicentre, Paris, France.; d World Health Organization, Geneva, Switzerland.; e World Health Organization, Lusaka, Zambia.

## Abstract

**Objective:**

To describe the implementation and feasibility of an innovative mass vaccination strategy – based on single-dose oral cholera vaccine – to curb a cholera epidemic in a large urban setting.

**Method:**

In April 2016, in the early stages of a cholera outbreak in Lusaka, Zambia, the health ministry collaborated with Médecins Sans Frontières and the World Health Organization in organizing a mass vaccination campaign, based on single-dose oral cholera vaccine. Over a period of 17 days, partners mobilized 1700 health ministry staff and community volunteers for community sensitization, social mobilization and vaccination activities in 10 townships. On each day, doses of vaccine were delivered to vaccination sites and administrative coverage was estimated.

**Findings:**

Overall, vaccination teams administered 424 100 doses of vaccine to an estimated target population of 578 043, resulting in an estimated administrative coverage of 73.4%. After the campaign, few cholera cases were reported and there was no evidence of the disease spreading within the vaccinated areas. The total cost of the campaign – 2.31 United States dollars (US$) per dose – included the relatively low cost of local delivery – US$ 0.41 per dose.

**Conclusion:**

We found that an early and large-scale targeted reactive campaign using a single-dose oral vaccine, organized in response to a cholera epidemic within a large city, to be feasible and appeared effective. While cholera vaccines remain in short supply, the maximization of the number of vaccines in response to a cholera epidemic, by the use of just one dose per member of an at-risk community, should be considered.

## Résumé

**Objectif:**

Rendre compte de la mise en œuvre et de la faisabilité d'une stratégie de vaccination de masse innovante – fondée sur un vaccin anticholérique oral à dose unique – pour enrayer une épidémie de choléra dans une vaste zone urbaine.

**Méthodes:**

En avril 2016, aux premiers stades d'une flambée de choléra à Lusaka, en Zambie, le ministère de la Santé a collaboré avec Médecins Sans Frontières et l'Organisation mondiale de la Santé pour organiser une campagne de vaccination de masse fondée sur un vaccin anticholérique oral à dose unique. Pendant 17 jours, ces partenaires ont fait appel à 1700 membres du personnel du ministère de la Santé et bénévoles communautaires pour mener des activités de sensibilisation des communautés, de mobilisation sociale et de vaccination dans 10 municipalités. Chaque jour, des doses de vaccins ont été livrées sur les sites de vaccination et une estimation de la couverture administrative a été effectuée.

**Résultats:**

En tout, les équipes de vaccination ont administré 424 100 doses de vaccins à une population cible estimée à 578 043 individus, ce qui se traduit par une couverture administrative estimée à 73,4%. Après la campagne, seuls de rares cas de choléra ont été signalés et aucun signe de propagation de la maladie dans les zones de vaccination n'a été noté. Le coût total de la campagne – 2,31 dollars des États-Unis ($US) par dose – incluait le coût relativement bas de la livraison locale – 0,41 $US par dose.

**Conclusion:**

L'organisation d'une campagne de vaccination réactive précoce, ciblée et à grande échelle – fondée sur un vaccin oral à dose unique – pour répondre à une épidémie de choléra dans une grande ville s'est avérée faisable et efficace. Alors que les vaccins anticholériques demeurent rares, il convient d'envisager la maximisation du nombre de vaccins pour faire face à une épidémie de choléra à travers l'utilisation d'une seule dose par membre d'une communauté vulnérable.****

## Resumen

**Objetivo:**

describir la aplicación y la viabilidad de una estrategia innovadora de vacunación masiva, basada en una vacuna oral contra el cólera de una sola dosis, para frenar una epidemia de cólera en un entorno urbano grande.

**Método:**

en abril de 2016, en las primeras etapas de un brote de cólera en Lusaka, Zambia, el Ministerio de Salud colaboró con Médecins Sans Frontières y la Organización Mundial de la Salud en la organización de una campaña masiva de vacunación basada en una vacuna oral contra el cólera de una sola dosis. Durante un período de 17 días, los socios movilizaron a 1700 voluntarios de personal sanitario y voluntarios de la comunidad para sensibilizar a la comunidad y para actividades de vacunación y movilización social en 10 municipios. Cada día, las dosis de la vacuna se entregaron en los lugares de vacunación y se estimó la cobertura administrativa.

**Resultados:**

en general, los equipos de vacunación administraron 424.100 dosis de la vacuna a una población objetivo de unos 578.043 habitantes, lo que resultó en una cobertura administrativa estimada del 73,4%. Después de la campaña, se registraron pocos casos de cólera y no hubo pruebas de propagación de la enfermedad dentro de las zonas vacunadas. El costo total de la campaña (2,31 dólares estadounidenses (USD) por dosis) incluyó el coste relativamente bajo de la entrega local (0,41 $ por dosis).

**Conclusión:**

se descubrió que una campaña reactiva temprana dirigida y en gran escala con una vacuna oral de dosis única, organizada en respuesta a una epidemia de cólera dentro de una gran ciudad, era factible y aparentemente efectiva. Si bien las vacunas contra el cólera siguen siendo escasas, se debe considerar la maximización del número de vacunados en respuesta a una epidemia de cólera, mediante el uso de una sola dosis por miembro de una comunidad en riesgo.

## ملخص

**الغرض:**

وصف لإجراءات تنفيذ استراتيجية مبتكرة للتطعيم الجماعي وإمكانية تطبيقها – إذ تقوم الاستراتيجية على تقديم جرعة واحدة من لقاح الكوليرا يتم تلقيها عبر الفم – وذلك للحد من انتشار وباء الكوليرا في منطقة حضرية واسعة النطاق.

**الطريقة:**

شهد عام 2016، مع المراحل الأولى لظهور مرض الكوليرا في مدينة "لوساكا" في زامبيا، تعاونًا بين وزارة الصحة بالبلاد من جهة ومنظمة أطباء بلا حدود (Médecins Sans Frontières) ومنظمة الصحة العالمية من جهة أخرى بشأن تنظيم حملة تطعيم جماعي تقوم على تقديم جرعة واحدة عبر الفم من لقاح الكوليرا. وخلال فترة تبلغ 17 يومًا، عملت الأطراف المتعاونة على حشد 1700 فرد من العاملين بوزارة الصحة والمتطوعين في الخدمات المجتمعية من أجل تحقيق التوعية المجتمعية والتعبئة الاجتماعية وتنفيذ أنشطة التطعيم في 10 أقاليم. وتم توفير جرعات التطعيم في كل يوم في مواقع التطعيم وجرى تقييم للتغطية المتعلقة بالجرعات المقدمة.

**النتائج:**

قدمت فرق التطعيم بوجه عام 424100 جرعة من اللقاح لمجموعة مستهدفة من السكان بلغ عددها 578043 فردًا حسب التقديرات، مما أثمر عن وجود تغطية للجرعات المقدمة تم تقديرها بنسبة ‏73.4%. وبعد الحملة، قل العدد الذي يتم الإبلاغ به من حالات الإصابة بالكوليرا، ولم يوجد أي دليل على انتشار المرض في المناطق التي تم فيها التطعيم. وكانت التكلفة الإجمالية للحملة – التي بلغت 2.31 دولارًا أمريكيًا للجرعة الواحدة، شاملةً التكلفة المنخفضة نسبيًا لتوفير الجرعة محليًا – قد بلغت 0.41 دولارًا أمريكيًا للجرعة الواحدة.

**الاستنتاج:**

تبين لنا أن تنفيذ حملة موجهة واسعة النطاق في مرحلة مبكرة للتصدي للمرض باستخدام جرعة واحدة من لقاح فموي – وهي الحملة التي تم تنظيمها لمواجهة وباء الكوليرا على نطاق مدينة كبرى – أمر يمكن تحقيقه وبدت له نتائج مجدية. وبالرغم من استمرار المعاناة من النقص في الموارد المتاحة للقاح الكوليرا، يجب النظر في تحقيق أقصى استفادة ممكنة من عدد اللقاحات المتاحة للتصدي لوباء الكوليرا، وذلك عن طريق استخدام جرعة واحدة فقط لكل فرد من أفراد المجتمع المهدد بالإصابة بالمرض. ****

## 摘要

**目标:**

旨在说明基于单剂量口服霍乱疫苗的创新型大规模接种策略的实施和可行性，以遏制大型城市环境中的霍乱疫情。

**方法:**

2016 年 4 月，在赞比亚卢萨卡霍乱爆发的早期阶段，卫生部门与无国界医生组织和世界卫生组织合作组织了基于单剂量口服霍乱疫苗的大规模疫苗接种活动。在 17 天的时间里，合作方共动员了 1700 名卫生部工作人员和社区志愿者在 10 个乡镇开展社区宣传、社会动员和疫苗接种活动。每天将疫苗剂量送到疫苗接种处并估计接种覆盖率。

**结果:**

总体而言，疫苗接种小组对估计的目标人群 578043 人进行了 424100 剂疫苗接种，估计接种覆盖率为 73.4％。此项活动后，报告的霍乱案例很少，没有证据表明疫苗接种区域内有传染病。活动的总费用——每剂量 2.31 美元，包括相对较低的本地交付成本（每剂量 0.41 美元）。

**结论:**

我们发现，使用单剂量口服疫苗的早期大规模针对性反应性活动是为了应对大城市内的霍乱疫情而组织起来的，可行且有效。尽管霍乱疫苗仍然供不应求，但每一名处于危险社区中的成员只需一个剂量，应对霍乱疫情所需的最大接种疫苗数量应该考虑在内。

## Резюме

**Цель:**

Описать реализацию и осуществимость инновационной стратегии массовой вакцинации, основанной на применении однодозовой пероральной противохолерной вакцины, для борьбы с эпидемией холеры в условиях крупных городов.

**Методы:**

В апреле 2016 года на ранних стадиях вспышки холеры в Лусаке, Замбия, Министерство здравоохранения сотрудничало с организацией «Врачи без границ» и Всемирной организацией здравоохранения при проведении кампании массовой вакцинации, основанной на применении однодозовой пероральной противохолерной вакцины. В течение 17 дней партнеры мобилизовали 1700 сотрудников Министерства здравоохранения и общинных добровольцев для повышения информированности населения, социальной мобилизации и вакцинации в 10 поселках. Ежедневно на участки вакцинации доставлялись дозы вакцины и оценивался административный охват вакцинацией.

**Результаты:**

В целом группами вакцинации были введены 424 100 доз вакцины в предполагаемой целевой популяции в количестве 578 043 человек, в результате чего административный охват оценивался как 73,4%. После кампании сообщалось о редких случаях заболевания холерой и не было признаков распространения болезни в вакцинированных районах. Общие расходы на кампанию составили 2,31 доллара США (US$) на 1 дозу, включая относительно низкую стоимость местной доставки — 0,41 доллара США на 1 дозу.

**Вывод:**

Мы обнаружили, что ранняя и крупномасштабная целевая реактивная кампания с применением однодозовой пероральной вакцины, организованная в ответ на эпидемию холеры в крупном городе, осуществима и эффективна. Поскольку по-прежнему имеется дефицит противохолерных вакцин, следует рассмотреть вопрос об увеличении числа доступных вакцин в ответ на эпидемию холеры за счет использования только одной дозы на одного члена сообщества, находящегося в зоне риска.

## Introduction

The World Health Organization (WHO) has estimated that there are 1.3–4 million cholera cases and 21 000–143 000 cholera-related deaths each year.^1^ Cholera is a poverty-related disease and large-scale cholera epidemics continue to occur in low-income countries. In 2015, for example, an outbreak that involved approximately 40 000 people affected parts of the Democratic Republic of Congo, Kenya and the United Republic of Tanzania.^2^ The prevention and control of epidemics are usually based on a multidisciplinary integrated approach that may include community sensitization, intensified epidemiological surveillance, improved access to clean water, hygiene and sanitation and the treatment of confirmed and suspected cases. Recent large-scale cholera outbreaks have shown the limitations of standard response measures and the need for improved strategies.^2^

The first documented campaign using oral cholera vaccine in a humanitarian context was carried out in 1997, in Uganda.^3^ Since then, similar preventive vaccinations have been organized in areas considered at risk of a cholera outbreak.^4^^–^^6^ In recent years, increased use of oral cholera vaccine in different settings and mostly in a preventive manner has provided evidence of the feasibility and effectiveness of vaccination campaigns against cholera.^7^^,^^8^ A turning point in the implementation of cholera vaccination campaigns was WHO’s prequalification of the Shanchol oral cholera vaccine in 2011.^9^ In 2013, under the supervision of the International Coordination Group on Vaccine Provision, a global stockpile of oral cholera vaccine was created to strengthen the capacity for action against cholera in emergency settings.^9^^,^^10^

Timely reactive vaccinations – i.e. vaccinations in response to an existing epidemic – for cholera outbreaks are challenging, because it can take considerable time to identify and report a cholera epidemic and it is hard to predict outbreaks. Other possible challenges are inadequate financial and human resources and inadequate number of doses to target everyone at risk of cholera, especially for a multi-dose campaign.^11^ While the licensed protocol requires two doses of oral cholera vaccine to be given two weeks apart, the feasibility of such a regimen has been, and remains, limited by the availability of suitable vaccines doses. Given a global shortage of such vaccines, a single-dose strategy, which, given a fixed number of doses, could cover twice as many people as a two-dose regimen, should be considered.^12^ This strategy was first used under field conditions in 2015, in Juba, the conflict-ravaged capital of South Sudan, when more than 160 000 people were vaccinated in response to a cholera outbreak.^11^ In a short-term observational study, the effectiveness of a single dose of vaccine in this campaign in Juba was estimated to be 87.3% (95% confidence interval, CI: 70.2–100.0).^13^ In 2014, a clinical trial in Bangladesh indicated that a single dose of oral cholera vaccine would give 40% (95% CI: 11–60) and 63% (95% CI: 24–82) protection against all and severe episodes of cholera, respectively.^14^

Cholera is a public-health problem in many areas of Zambia, but is a particular problem in the capital, Lusaka.^15^ Although there were annual cholera epidemics in Zambia between 2003 and 2011, no confirmed cases of the disease were reported in the country in 2012–2015. When, therefore, a few people with the disease were detected in Lusaka in February 2016, general levels of immunity to cholera were assumed to be relatively low in Zambia. At the time, there was concern that there was considerable risk of an imminent major outbreak. The Lusaka District Health Office quickly organized a response according to the national guidelines on cholera control.^16^ In addition, the Zambian Ministry of Health, with support from Médecins Sans Frontières and WHO, implemented a reactive vaccination campaign with the aim of stopping transmission of *Vibrio cholerae* in Lusaka. In 2016, almost 600 000 people were living in the nine townships of Lusaka that were considered at greatest risk, because they had been the foci of cholera outbreaks in the previous two decades. Given the large target population and the global shortage of appropriate vaccine doses, it was decided to use a single-dose vaccination campaign, rather than a two-dose strategy, and so allow the largest number of vulnerable people to be vaccinated with the doses that were available.

Here we describe the context of the interventions and the decision-making process and we evaluate the feasibility of conducting such a large-scale reactive campaign of cholera vaccination in high-risk and densely-populated urban areas.

## Methods

### Setting

Lusaka is a fast-growing city with a population of over 2 million people. Between 2003 and 2011, there were annual cholera epidemics, 26 000 cholera cases and 860 cholera-related deaths recorded in the city.^15^^,^^17^ Over this period, Médecins Sans Frontières helped the health ministry to control the cholera outbreaks in the city and record the numbers of people with cholera per township, the seasonal pattern of cholera and assess water and sanitation quality in each township.^17^ Most epidemics recorded in the city since 2003 began in December/January and finished, as the rainy season ended, around April/May.^17^ They started in one or more of nine townships that had particularly poor water supplies and drainage systems.^17^

**Fig. 1:**
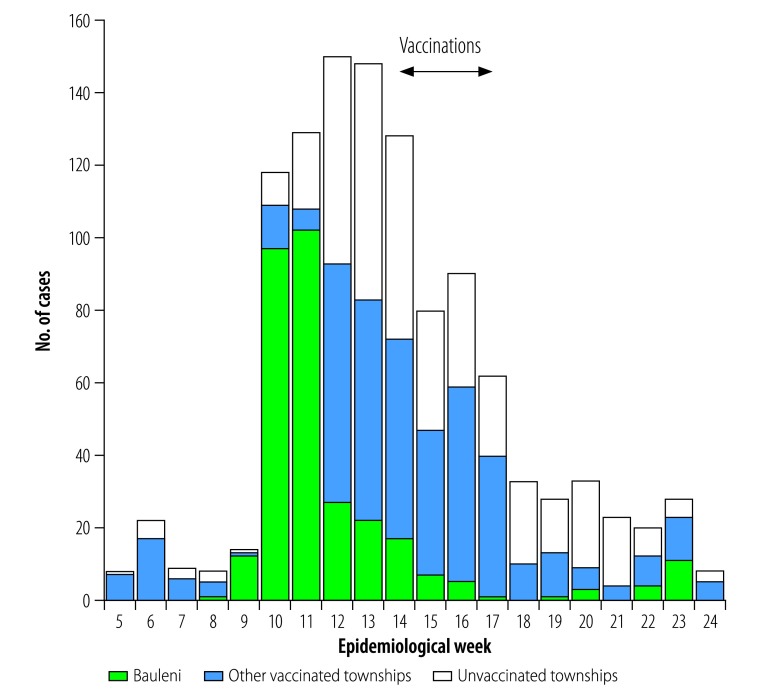
Weekly numbers of confirmed and/or suspected cases of cholera reported in Lusaka, Zambia, 2016

Notes: The graph shows epidemiological weeks. Single doses of oral cholera vaccine were administered between week 14 and week 17. The case numbers for Bauleni township, the other nine townships that were targeted in a mass immunization campaign based on single doses of oral cholera vaccine and the city’s other townships, in which there were no immunizations with oral cholera vaccine, are presented separately.

On 4 February 2016 – i.e. in epidemiological week 5 of 2016 – a person with cholera was identified in Lusaka. This represented the first confirmed case in the city since 2011. Confirmed and/or suspected cases were identified over the next few days and a cholera outbreak was declared. Laboratory analysis showed that the agent causing this outbreak was *V. cholerae* O1 El Tor-Ogawa. The number of confirmed and/or suspected cases increased rapidly, from 14 in epidemiological week 9 to 118 in epidemiological week 10 (Fig. 1).

### Decision-making and vaccine provision

In early March 2016, the health ministry, fearing an imminent major epidemic, requested the assistance of Médecins Sans Frontières in controlling the epidemic. There were 277 confirmed and/or suspected cholera cases reported on 18 March. Of these cases, 57 were found positive in a rapid diagnostic test for cholera and 25 were confirmed by culture. As a result of these observations, the health ministry, Médecins Sans Frontières and WHO agreed to add vaccination to the standard cholera-control activities that had already been implemented. However, when this agreement was made, the global emergency stockpile of oral cholera vaccine, containing about 1.3 million doses, was far too small to allow Zambia and several other countries with urgent requirements for cholera vaccine to give everyone in at-risk populations two doses. We therefore decided to follow a single-dose vaccination strategy in Lusaka, to cover everyone who was older than one year and lived in a high-risk township, and to consider delivering a second dose later, when more vaccine doses became available. On March 24, the health ministry sent a request to the International Coordination Group, to access 598 131 doses from the emergency stockpile^18^ and they received a positive response five days later. The allocated vaccine was Shanchol (Shantha Biotechnics, Hyderabad, India) and the vaccine doses arrived in Zambia, in two shipments, on 7 and 8 April. Although Shanchol was not registered in Zambia, the health ministry approved its emergency use and facilitated the importation process.

### Target population

The target population included all individuals older than one year who lived in one of the 10 townships of Lusaka that, in March 2016, were considered high-risk for cholera. Nine townships had the highest attack rates in the epidemics in 2003–2011 and one township had a high incidence of suspected cholera when the vaccine request was sent. To estimate the population of these townships, we combined population projections from the 2010 national census^19^ with other relevant population data (Lusaka City Council, unpublished data, 2016).

### Vaccination strategy

**Fig. 2:**
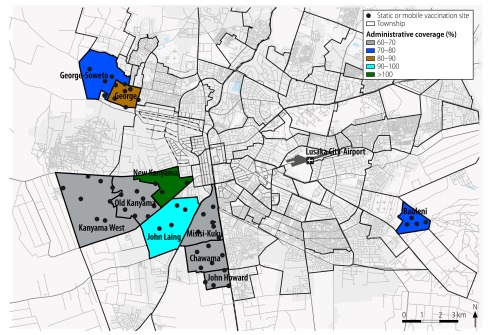
Estimated administrative coverages in the 10 townships of Lusaka targeted in a mass immunization campaign against cholera, Zambia, 2016

Note: Data are shown only for the areas targeted in a mass immunization campaign based on single doses of oral cholera vaccine, i.e. Bauleni township in the east of the city and nine other townships in the west.

Vaccination teams implemented a two-phase strategy designed to cover the entire target population within a short period. The first phase involved the setting up of static vaccination sites, which were easy to organize and control, in health facilities or churches or schools. Each static site was covering a circular area, centred on the site, with a maximum radius of 500 m (Fig. 2). Each such site was scheduled to open from 07:00 to 18:00 on each of two to six days, the exact period depending on the turn-out of the target population. We set a vaccination target of each static site to 5500 doses per day, to match the financial and logistical resources that were available.

The second phase comprised a three-day catch-up campaign in which mobile vaccination teams operated, on main roads or in markets or other socially active places, to vaccinate targeted individuals who had failed to attend the static sites.

### Vaccination teams

Each vaccination team was composed of 23 volunteers and a qualified nurse from the health ministry who acted as team leader. Before the campaign, we organized a half-day training session in each high-risk township. This included a practical exercise in organizing a vaccination site. The team composition was such that, at each vaccination site, two vaccination lines, each run by two vaccinators, two people preparing vials, four recorders filling in cards and one person summarizing the records on a tally sheet, could be formed. Other team members were responsible for crowd control. In an attempt to facilitate the acceptance of the campaign, we selected the team members for each targeted township from the local population. Overall, 989 individuals created 53 teams, 42 at static sites and 11 running the mobile sites. At the height of the activity there were 17 sites working simultaneously.

### Social mobilization

In each targeted township, qualified environmental health technicians from the health ministry organized three days of community sensitization and mobilization before the vaccinations began and then continued similar activities for the duration of the campaign. A flyer explaining the characteristics of the vaccine and the dates and locations of the vaccination sites was developed locally and handed out. Vehicles with sound systems were driven around the targeted townships to advertise the vaccinations. Awareness-raisers worked near the vaccination sites. There were another 745 individuals, again a mix of health ministry staff and community volunteers, involved in the social mobilization.

### Cold-chain management

The Universal Child Immunization’s Secretariat Office in Lusaka stored the vaccines in a cold room. We distributed the vaccines to vaccination sites, with icepacks, as 1500-dose boxes in their original packaging. The vaccines were used at ambient temperature on the vaccination day. The vaccinators checked the vaccine vial monitor before administering each dose.

### Data collection and analysis

Recorders categorized the age of each vaccinee as one to four years, five to 14 years or adult, i.e. at least 15 years, on a tally sheet, but ignored sex. On a daily basis, the recorders compiled the sheets to allow the daily evaluation of administrative coverages to be estimated each day. The estimated coverages were then used to plot a performance map for each targeted township. By rapidly sharing such maps among partners, it was possible to make quick compensations for poor coverage in particular areas, e.g. increasing the duration of the vaccination period at some sites and reinforcement of sensitization activities. We calculated vaccine wastage as the percentage of the number of doses leaving the cold room that, according to the tally sheets, were lost. We expressed cholera attack rates as the numbers of confirmed and/or suspected cases per 10 000 people and we used Poisson regressions to compare such rates by age group and sex.

Although we assessed the short-term effectiveness of the single-dose strategy and we implemented a coverage survey, the results of these activities will be reported separately.

### Ethics

The vaccination campaign was conducted as part of the public health response to the cholera outbreak and was approved by the Zambian Ministry of Health.

## Results

### Administrative coverage

Between 9 and 25 April 2016, 424 100 vaccine doses were administered to an estimated combined target population of 578 043, giving an estimated administrative coverage of 73.4%. Estimated administrative coverage in the targeted townships varied between 61.9% and 105.8% (Fig. 2).

Of the doses that vaccination teams administered, 371 279 (87.5%) were administered during the first phase – i.e. at the static sites – and the rest during the catch-up at mobile sites (Table 1). Although most of the doses used in the catch-up phase were used on adults, estimated administrative coverage remained generally higher in children than in adults (Table 1).

**Table 1:** Numbers of vaccines in each age group and each phase of the single-dose vaccination campaign against cholera and overall administrative coverages, Lusaka, Zambia, 2016

Age (years)	Target population^a^	No. of vaccines (% in age group)			Administrative coverage, %
		First phase	Catch-up	Total	
1–5	78 189	75 725 (92.0)	6609 (8.0)	82 334	105.3
5–15	144 652	163 612 (91.7)	14 839 (8.3)	178 451	123.4
> 15	355 202	131 942 (80.8)	31 373 (19.2)	163 315	46.0
All	578 043	371 279 (87.5)	52 821 (12.5)	424 100	73.4

^a^ Estimates based on the 2016 projection of the results of the 2010 national census.

### Performance indicators

At each of the static sites, the vaccination team administered cholera vaccine to a mean of 2256 (range: 306–5843) people per day, i.e. to about half of the target of 5500. The corresponding number for the mobile sites was even lower: 1492 (range: 371–3757).

Vaccine wastage was less than 0.01% (459/424 100). At the end of the campaign, 174 031 doses remained in the cold room and were retained for possible use as second doses. There were no reports of doses being discarded because of invalid vaccine vial monitors.

### Costs

Of the total cost of the vaccination campaign, which was 978 614 United States dollars (US$), US$ 784 831 (80.2%) were used on vaccine procurement (Table 2). Local delivery cost was US$ 173 677, that is, a mean of just US$ 0.41 per dose administered.

**Table 2:** Costs of the single-dose vaccination campaign against cholera, Lusaka, Zambia, 2016

Characteristic	Costs, US$ (% of total)
Vaccine purchase^a^	784 831 (80.2)
International shipment^a^	20 106 (2.0)
Staff incentives^b^	65 922 (6.7)
Food, identification cards and other staff expenses^b^	10 543 (1.1)
Vaccination equipment and consumables	25 325 (2.6)
Social mobilization materials and consumables	15 252 (1.6)
Logistical costs of venue use and waste management	25 081 (2.6)
Transport costs	31 554 (3.2)
Total cost of the campaign^c^	978 614 (100)
Total local delivery cost^c^	173 677 (17.7)

US$: United States dollars.

^a^ The proportion of the total costs, for the 598 131 imported doses, represented by the 424 100 doses that were administered in the single-dose campaign.

^b^ Excluding the administrative costs for, and salaries of, any overseas personnel.

^c^ Excluding the costs of monitoring and evaluation.

### Outbreak characteristics

During the entire 2016 cholera outbreak in Lusaka, which ran between epidemiological week 5 and week 24, the health ministry reported 1139 confirmed and/or suspected cholera cases (Fig. 1). Over the same period, the ministry recorded 20 cholera-related deaths, of which 10 occurred in community settings and 10 in health facilities. These 20 deaths represent 1.76% of the confirmed and/or suspected cholera cases that were reported. The weekly incidence of cases peaked in epidemiological week 12, when 150 suspected cases were reported. In Lusaka, the overall attack rate was 4.89 per 10 000 people. Attack rates were significantly higher among children younger than five years than among older individuals: 5.80 vs 4.71 per 10 000 people (attack rate ratio: 1.23; 95% CI: 1.06–1.42). They were also significantly higher among males than among females: 5.20 vs 4.59 per 10 000 people (attack rate ratio: 1.13; 95% CI: 1.01–1.27).

Although we only implemented the vaccination campaign after the epidemic peaked in the Bauleni township, the campaign preceded the corresponding peaks in the other nine targeted townships (Fig. 1).

### Discussion

In 10 apparently high-risk townships in Lusaka the vaccinators administered over 420 000 doses of oral cholera vaccine, resulting in over 70% of the target population being vaccinated. Box 1 summarizes the major achievements and lessons learnt from the campaign. Such mass campaigns appear to be feasible within large urban settings. When, in 2016, the health ministry found itself faced with the threat of a major outbreak of cholera in Lusaka, it had no previous experience with vaccination campaigns against the disease. However, after careful review of the available literature, particularly the then-unpublished results of the single-dose reactive campaign organized in South Sudan^11^^,^^13^ the health ministry took the decision to use a single-dose strategy. This was because insufficient vaccine doses were available to follow the more usual, two-dose regimen. The possibility of delivering a second dose at a later stage, once adequate doses of vaccine became available, was discussed during the planning phase. Using a combination of new stock and the doses leftover from the single-dose campaign, the health ministry administered second doses on 16–21 December 2016, i.e. before the 2016/2017 rainy season. Since then, single-dose campaigns based on oral cholera vaccine have taken place in Haiti and Mozambique.^20^ In April 2017, WHO’s Strategic Advisory Group of Experts on immunization updated its recommendations on the use of oral cholera vaccine, stating that “a single dose strategy could be considered in areas experiencing cholera outbreaks. Considering the limited evidence about the duration of protection, additional doses might be needed to ensure longer-term protection.”^21^

Box 1 **Major achievements and lessons learnt from Lusaka vaccination campaign, Zambia, 2016**

**Outcome**

- In response to cholera outbreaks, large targeted reactive vaccination campaigns are feasible in large urban settings and can be deployed in a timely manner.
- Given a global shortage of cholera vaccines, a single-dose strategy allows a greater population to be vaccinated. In Lusaka, such a strategy allowed targeting of the entire population in the areas considered most at risk of *Vibrio cholerae* transmission.
- The per-dose cost of the Zambia campaign, implemented in response to an outbreak, was at least as low as that of campaigns implemented in non-outbreak settings and, since vaccination teams were underused, this could have been reduced.

**Implementation**

- Factors that allowed a relatively quick decision-making process were: good understanding of the local cholera epidemiology, rapid confirmation and declaration of the outbreak by the health ministry and good collaboration between Médecins Sans Frontières, the health ministry and the World Health Organization in sending the vaccine request to the International Coordination Group early. In addition, the health ministry’s anticipation of regulatory hurdles was a key factor in ensuring that the first use of oral cholera vaccine in Zambia began quickly.
- Use of static vaccination sites was probably a more efficient and cheaper strategy than house-to-house visits.
- Age stratification of the tally-sheet data allowed the catch-up activities to be focused mainly on adults, i.e. the age group that had relatively poor coverage in the first phase of the campaign. The recording on the tally sheets of the sex of each vaccine could have improved such focus on specific low-coverage groups. However, the coverages estimated in most urban campaigns of cholera vaccination indicate that specific approaches to improve coverage among adults, particularly men, should probably be implemented from the onset of any such campaign.
- In Lusaka and, probably, similar large urban settings, the cold chain for an oral cholera vaccine with good thermostability can be simply based on a single cold room for the time of the vaccination campaign.

In reactive mass immunizations, a speedy response is needed if a major outbreak is to be prevented. In Lusaka in 2016, mass immunization began just 21 days after the decision to vaccinate was made and two months after the appearance of the first reported case. A key step was the rapid detection and declaration of the outbreak by the health ministry. Knowledge of the local cholera epidemiology and good collaboration between the ministry and its international partners allowed the choice of vaccination strategy, the preparation of the request for the vaccine doses from the global stockpile and the import of the released doses to proceed smoothly. This process also allowed vaccinations to begin as soon as the doses reached Lusaka. The cold-chain logistics and vaccine distribution in Lusaka were simpler than those described in some other campaigns,^7^ thanks, in part, to the thermostability of the vaccine used.

Although some campaigns based on oral cholera vaccine have reached higher coverages, the overall administrative coverage achieved in Lusaka (73.4%) was similar to that recorded in other urban campaigns.^11^^,^^22^^,^^23^ The accuracy of data on administrative coverage is often limited by the use of population estimates, especially when such estimates relate to a large city, such as Lusaka, that has a dynamic population and many informal settlements. During the 2016 vaccination campaign described here, there were xenophobic riots in some of the targeted townships^24^ and this unrest may have contributed to a lower coverage than initially expected. In Lusaka in 2016, as in several previous vaccination campaigns against cholera,^6^^,^^11^^,^^23^^,^^25^ administrative coverage was much poorer among adults than among children (Table 1). Although vaccinee sex was not recorded on the tally sheets, the vaccination teams reported that they vaccinated far fewer men than women at the static sites. While the catch-up campaign made it possible to narrow the gap in coverage between adults and children, coverage among adults, and particularly among men, remained relatively poor. In future similar campaigns, the tailoring of catch-up specifically to benefit women and, particularly, men needs to be carefully considered.

As the mean number of people vaccinated daily at each static site was considerably lower than the set target number, vaccination teams at the sites were generally underused. There was probably, therefore, scope to reduce the mean per-dose cost of the local implementation of the campaign. However, even with the underuse, the mean per-dose cost of local delivery in Lusaka (US$ 0.41) accounted for less than 20% of the total per-dose costs and was lower than the corresponding values previously reported for the Shanchol vaccine. For example, it was lower than the corresponding values reported in India in 2011 (US$ 0.49),^26^ Bangladesh in 2011 (US$ 0.76),^23^ Guinea in 2012 (US$ 0.89),^27^ and South Sudan in 2014 (US$ 0.63).^28^ Between-study comparisons of per-dose costs have to be made with caution, because of the differences in the collection of cost data, estimation of per-dose costs and value of the United States dollar.^29^

The number of people reported with cholera in Lusaka after the single-dose campaign remained limited and the outbreak did not spread within any of the 10 targeted townships even when access to safe drinking water and sanitation remained poor.

This large-scale single-dose campaign, run in a densely populated urban area, showed to be feasible and appeared effective. Given the continuing global shortage of cholera vaccines, the use of single doses of oral cholera vaccine will remain an important alternative vaccine-delivery strategy in future cholera epidemics.
